# Wildfire Suppression Costs for Canada under a Changing Climate

**DOI:** 10.1371/journal.pone.0157425

**Published:** 2016-08-11

**Authors:** Emily S. Hope, Daniel W. McKenney, John H. Pedlar, Brian J. Stocks, Sylvie Gauthier

**Affiliations:** 1 Great Lakes Forestry Centre, Canadian Forest Service, Natural Resources Canada, Sault Ste. Marie, Ontario, Canada; 2 BJ Stocks Wildfire Investigations Ltd., Sault Ste. Marie, Ontario, Canada; 3 Laurentian Forestry Centre, Canadian Forest Service, Natural Resources Canada, Sainte-Foy, Québec, Canada; University of Guelph, CANADA

## Abstract

Climate-influenced changes in fire regimes in northern temperate and boreal regions will have both ecological and economic ramifications. We examine possible future wildfire area burned and suppression costs using a recently compiled historical (i.e., 1980–2009) fire management cost database for Canada and several Intergovernmental Panel on Climate Change (IPCC) climate projections. Area burned was modelled as a function of a climate moisture index (CMI), and fire suppression costs then estimated as a function of area burned. Future estimates of area burned were generated from projections of the CMI under two emissions pathways for four General Circulation Models (GCMs); these estimates were constrained to ecologically reasonable values by incorporating a minimum fire return interval of 20 years. Total average annual national fire management costs are projected to increase to just under $1 billion (a 60% real increase from the 1980–2009 period) under the low greenhouse gas emissions pathway and $1.4 billion (119% real increase from the base period) under the high emissions pathway by the end of the century. For many provinces, annual costs that are currently considered extreme (i.e., occur once every ten years) are projected to become commonplace (i.e., occur once every two years or more often) as the century progresses. It is highly likely that evaluations of current wildland fire management paradigms will be necessary to avoid drastic and untenable cost increases as the century progresses.

## Introduction

Wildfires are a natural process across much of Canada’s 400 million hectares of forest ecosystems. They can threaten societal interests such as human health, forestry operations and timber values, residential and commercial property, and transportation and energy infrastructures. As such, considerable resources are spent on wildfire suppression by resource management agencies [1]. Over the 1970 to 2009 period, annual suppression costs ranged from $216 million (in 2009 Canadian dollars), to over $1 billion [2] with an average value of $537 million. This wide range in costs has been related to variation in weather, fuel conditions, area burned, and other operational factors [3–5].

Evolution in climate over the next several decades can be expected to add to the challenges. Projections indicate that northern regions, such as Canada, are likely to warm relatively more than other parts of the planet [6]; in fact, warming trends have already been documented in many areas of the country [7–9]. Several studies have examined the potential impacts of a changing climate on wildland fire regimes globally [10, 11] and in Canada [12–17]. The primary conclusion of these studies is that fire activity is likely to increase, albeit heterogeneously, across the country as climate changes. For example, Boulanger *et al*. [17] reported that annual area burned will increase by 1.5–4 times across the country by the end of the century; similarly, Flannigan *et al*. [18] reported an increase of 1.7–2.2 times over the same period. Fire season length in Canada is also forecast to increase by approximately 30 days over this period [19]. Studies in the United States have come to similar conclusions; fire seasons are likely to lengthen by two to three months in southern regions [20], and area burned is forecast to rise anywhere from 54 to 78%, depending on location [21, 22].

Suppression costs are expected to rise as fire activity intensifies [13, 23–25]; however, few studies have quantified this relationship. de Groot *et al*. [26] developed a regression model that related suppression costs to area burned over the 1970–1995 period and projected a 25–45% increase in suppression costs across Canada by the 2080–2100 period. McAlpine [27] examined Ontario fire suppression costs over a period from 1976–1998 to determine if costs, calculated as a result of fire characteristics, were increasing over time; the results suggested that costs were not impacted by climate change at that time. Detailed analyses of suppression costs under climate change are generally lacking for other jurisdictions as well. For example, suppression costs in the United States are broadly forecast to rise as a result of increased expansion of the wildland-urban interface and climate change [28–30]. Australian researchers forecast increased fire activity [31, 32] and have recognized the need to re-evaluate suppression budgets [32, 33].

A major reason for the lack of wildfire economics in Canada has been a scarcity of cost data to allow for such analyses. This situation has been at least partly addressed by a recent compilation of fire suppression cost data from across Canada [2]. Stocks [34] reported a 176% increase in 10 year average annual national costs over the 1970 to 2010 period. This increase was accompanied by a 177% increase in the 10 year average annual area burned over the same timeframe. Fire management agencies have attributed the cost increases to several factors, including the rate of inflation for key fire management components such as fuel and aircraft operations, increasing costs related to resource sharing across jurisdictions, and an increase in the number of people living in wildland-urban interface areas.

This wildfire suppression cost dataset, in combination with other data including spatial climate models [35, 36] and a spatially explicit delineation of protection zones within individual provinces [37], offers new opportunities to address questions related to forest fire economics. Here we employ these data to examine Canadian wildfire suppression costs under an evolving climate. We examine the relationship between historical costs, area burned, and a broad-scale climate moisture index that quantifies seasonal climatic dryness. We then project these relationships into the future using two emissions scenarios from four GCMs and discuss implications and limitations related to our findings.

## Materials and Methods

### Climate

Previous studies have reported success at modelling area burned as a product of broad-scale climate metrics [38]. Here we employ a climate moisture index (CMI; [39]) that combines temperature and precipitation data into a climate-based measure of dryness. CMI was available at a high resolution across the country, and has been shown to be a good indicator of drought-related impacts on tree mortality and distribution [39, 40], which has clear implications for regional fire regimes [41, 42]. We recognize that other climate-based metrics have been used for this purpose. One common approach has been to use outputs from the Canadian Fire Weather Index System [15, 18, 26], which typically requires inputs of daily weather data (e.g. precipitation, temperature, relative humidity, and wind speed) [15]. However, processing such data at the national scale for past and future years was beyond the computing capacities available for the current work. Furthermore, daily sequences of GCM-based precipitation estimates have been shown to be unreliable for predicting drought and flood-related events [43, 44]. For these reasons, we were interested in exploring the value of CMI, a relatively simple robust monthly metric of dryness for modelling annual area burned.

A gridded dataset of monthly and annual CMI values was generated as part of an ongoing effort to produce spatial climate data for North America (see [35] for details). Briefly, CMI was calculated at climate stations across Canada for each year over the period of interest by subtracting monthly potential evapotranspiration (PET) from monthly precipitation. Monthly PET values were calculated using a simplified Penman-Monteith equation, which requires only mean monthly maximum and minimum temperature as detailed in [39, 40]. Climate station CMI values were spatially interpolated using tri-variate thin plate smoothing splines (ANUSPLIN; [45]). For the current work, these models were mapped using a digital elevation model (DEM) at a 0.0833 arc second resolution (~10km).

Future estimates of CMI were derived from monthly temperature and precipitation projections made by four GCMs—CanESM2, CESM1CAM5, HadGEM2-ES, and MIROC-ESM (see http://cmip-pcmdi.llnl.gov/cmip5/docs/CMIP5_modeling_groups.pdf for full descriptions)–under two representative concentration pathways (denoted RCP 2.6 and 8.5; [46]). These RCPs generally bound current thinking on low and high greenhouse gas emissions over the course of the coming century [6] although emissions are currently tracking slightly above RCP 8.5 levels [47]. Raw GCM outputs were downscaled using an approach that involved adding coarse-scale changes (or deltas) predicted by the GCM to 1961–1990 climate normals at climate stations across North America (see [48] for details). A composite (i.e., average) projection was also calculated from the four individual GCMs for each RCP scenario.

**Fig 1:**
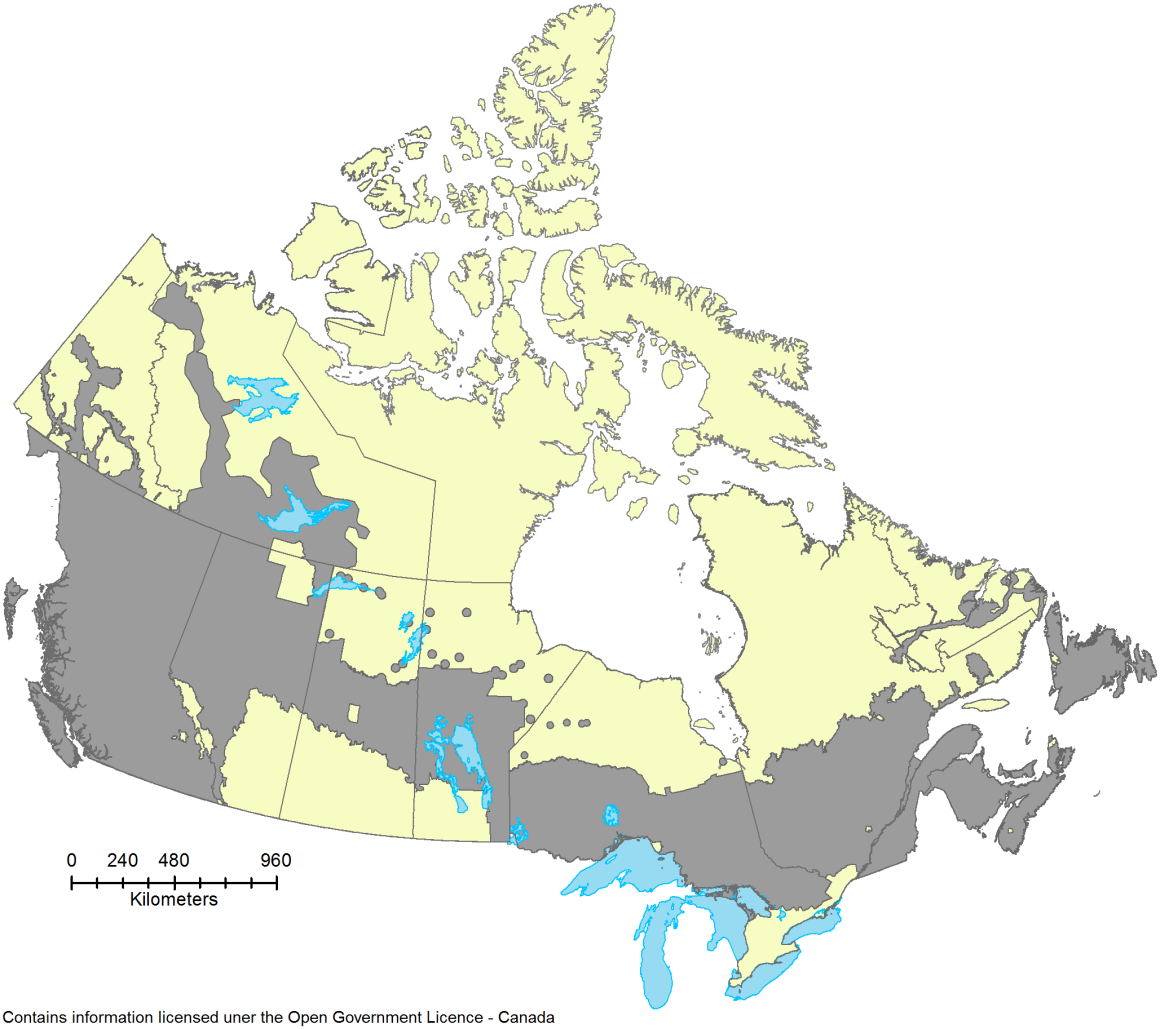
A map of the area examined. The grey shaded regions represent the forested area within the provincial fire suppression zones.

During preliminary analyses, we explored numerous temperature, precipitation, and CMI summaries (from annual to monthly) as explanatory variables, but found consistently strong relationships using a 4-month sum of CMI that spanned May to August—a period that currently coincides with the majority of the fire season across much of the country [18, 49]. Comparisons to other drought-related indices were not explored. For the analyses described below, a spatial average of this seasonal CMI was generated for each provincial fire suppression zone (Fig 1) in each year of the study.

### Area burned

Area burned data were obtained from the Natural Resources Canada National Fire Database, which contains fire-related data from provincial and territorial fire management agencies [50]. These data were limited to include only fires occurring in areas of fire suppression as defined by each province [37]. This focused the analysis on fires that would likely receive some level of fire suppression, and thus account for the majority of expenditures. It was assumed that suppression zones remained static throughout the time period examined. Data records were further limited to include only fires that occurred over the May—August period in each year; this time period captured more than 90% of the area burned in most provinces and further focused the analysis on the season when climate-fire relationships are expected to be strongest.

Due to missing area burned data, the Maritime Provinces (Nova Scotia, New Brunswick, Newfoundland and Labrador, and Prince Edward Island), the territory of Nunavut, and all national parks were excluded from the analysis. However, analysis of the fire cost data (described below) indicated that the combined annual fire suppression costs of the Maritime provinces accounted for only 5% of total national costs on average. Furthermore, the relatively wet conditions that prevail in this region of the country are projected to continue into future time periods, regardless of the greenhouse gas emissions scenario or GCM examined. Thus, the majority of present and future fire costs appear to be accounted for by the provinces and territories included in the analysis.

### Suppression costs

As noted, annual costs associated with fire suppression over the 1980–2009 period were obtained from a recently assembled dataset [S1 Table] which represents a significant improvement over historical suppression cost information in Canada. This dataset combined previously-published annual expenditure data for the 1970–1999 period, with additional 2000–2009 data compiled from a survey of all fire management agencies conducted by Stocks and Martel [2]. We limited our analysis to the 1980–2009 period, during which fire suppression zones were well defined; additionally, area burned estimates were considered less reliable during the 1970s due to limited fire monitoring capacities in remote areas at that time.

Costs are reported in 2009 Canadian dollars, and have been decomposed from total costs into fixed and variable costs. Fixed costs are defined as ongoing agency expenses, including infrastructure (e.g., buildings, equipment, and aircraft), maintenance and full-time staffing costs, while variable costs represent the costs directly associated with fire suppression activities, including seasonal salaries and additional firefighting equipment [34]. Though variable through time, fixed and variable costs have contributed approximately equally to total fire suppression costs during the 1970–2009 period [2]. Here we examine variable and fixed costs separately; variable costs are most likely to respond to climate driven variation in the fire regime, while fixed costs are assumed to be primarily a function of historical fixed costs [26].

### Analytical approach

In the first stage of the analysis, we developed a statistical model for each province that quantified the relationship between area burned and CMI. These analyses made use of the annual provincial estimates for each variable over the 1980 to 2009 period described above. The statistical relationship, modelled using linear regression, can be denoted as:

$$ln\text{(}Y_{i})\;=\;\beta_{0}+\;\beta_{1}CMI_{i}\;+\;\varepsilon_{i}$$ (1)

where *Y*_*i*_ represents the natural log of the yearly area burned in year *i*, *β*_*0*_ is the intercept value, *β*_*1*_ is the slope parameter, *CMI*_*i*_ is the CMI value in year *i*, and *ε*_*i*_ is the model error.

The second stage of the analysis quantified the relationship between area burned and variable cost over the same time period. This relationship was also modelled using linear regression and can be denoted as:

$$ln(Z_{i})=\beta_{0}+\beta_{1}ln(Y_{i})+\varepsilon_{i}\;$$ (2)

where *Z*_*i*_ is the natural log of the variable cost of fire suppression in year *i*; *β*_*0*_ is the intercept value, *β*_*1*_ is the slope parameter, *Y*_*i*_ is the natural log of the area burned in year *i*, and *ε*_*i*_ is the model error.

Fixed suppression costs were modelled using autoregressive techniques, such that current-year costs were modelled as a function of costs in previous years (similar to [26]). Specifically, each province’s time series of fixed costs were analyzed using an autoregressive integrated moving average (ARIMA) approach. Inspection of autocorrelation plots and related test statistics were used to determine the best model for each province, which included both simple autoregressive models (i.e., AR models) and, for provinces exhibiting trends in fixed costs through time, random walk with drift models [51]. While factors beyond historical fixed costs (such as technological or policy shocks) play a role in the current year’s fixed suppression costs, the modelling and forecasting of these factors was outside the scope of this analysis.

Each provincial linear regression model was tested for heterogeneity, independence, and normality through visual inspection of the model residuals. As a result, variable cost and area burned variables were natural log transformed to correct for significant skewing in the raw data. We further tested the variable cost data for evidence of temporal autocorrelation which, if present, could lead to inflated test statistic values for significance testing. Inspection of autocorrelation and partial autocorrelation functions indicated that this effect was negligible. All analyses were carried out using the R statistical software package [52].

Using future CMI projections described above in conjunction with eq (1), area burned was estimated for each province and year over the 2010–2100 period. Initial estimates of area burned were unrealistically high (i.e., larger than the study area for some provinces in some years); thus we introduced an area constraint based on the low probability of a stand re-burning before reaching 20 years of age (i.e. a 20 year minimum fire return interval) [53, 54]. A province’s ‘burnable area’ was calculated by subtracting the area burned over the preceding 20-year period from the total area covered by the province’s suppression zone. We also explored values of 10 and 30 years for this minimum fire return interval parameter to account for potential spatial variation and uncertainty in return intervals. Yearly area burned estimates were then taken as the minimum of the value generated by eq (1) or the burnable area. These area burned estimates were used in conjunction with eq (2) to obtain estimates of future annual variable suppression costs for each province. Forecasts of fixed suppression costs were added to projected variable costs to generate total cost estimates. These provincial-level results were summed to obtain national-scale projections of area burned and suppression costs.

## Results

### Historical and future CMI

**Fig 2:**
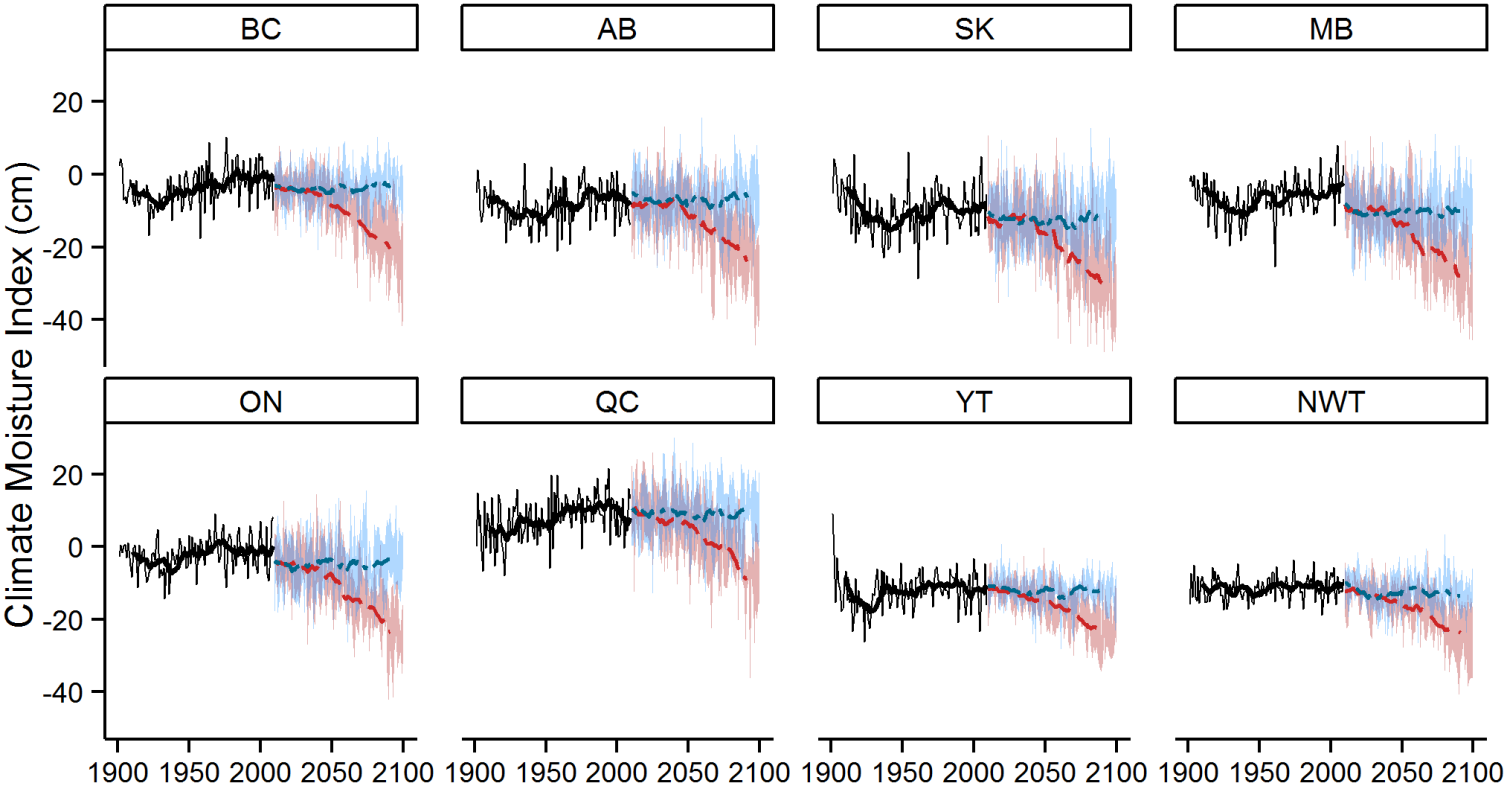
Historical and future CMI values under RCP 2.6 and 8.5 for all GCMs. The blue and red shaded regions (RCP 2.6 and RCP 8.5 respectively) reflect the yearly maximum and minimum CMI estimates under the four GCMs; the model average is represented by the blue (RCP 2.6) or red (RCP 8.5) dashed line. A ten year historical moving average from 1901 to 1979 is represented by the solid black line.

CMI exhibits considerable variation over both past and future time periods (Fig 2). Historically, most provinces show a decline (i.e. an increase in dryness) in the 4-month CMI during the early part of the 20^th^ century, followed by an increase until approximately 1990 when CMI declines again. Note that early 20^th^ century CMI estimates for far northern regions (e.g., the Northwest Territories) are uncertain due to the limited number of weather stations in these regions at that time. Under RCP 2.6, the average future CMI estimate from the four GCMs remains relatively stable throughout the current century. Under RCP 8.5, there is a strong decline in CMI in most provinces starting in approximately 2040 and continuing to the end of the century. Of particular note is the significant variation in CMI associated with the different GCM outputs (indicated by the range of blue and red values provided for each future year in Fig 2). If realized, such variations imply that, even under RCP 2.6, levels of dryness that are unprecedented in the recent historical record could be reached in most provinces during the first half of the 21^st^ century.

### Area burned and cost models

**Fig 3:**
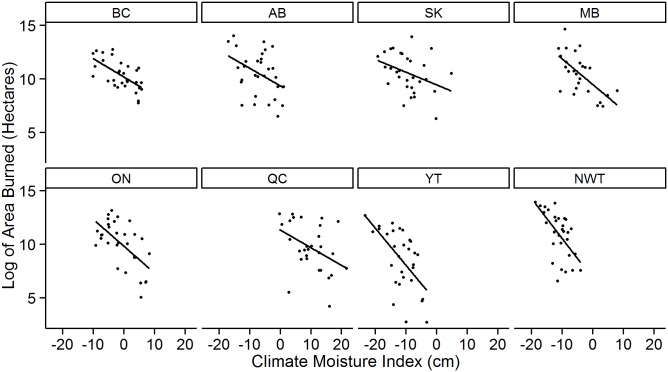
The provincial relationship between the sums of the monthly CMI values from May to August and area burned within the same time period. A positive CMI reflects an increase in moisture content, and a negative CMI reflects a decrease.

**Fig 4:**
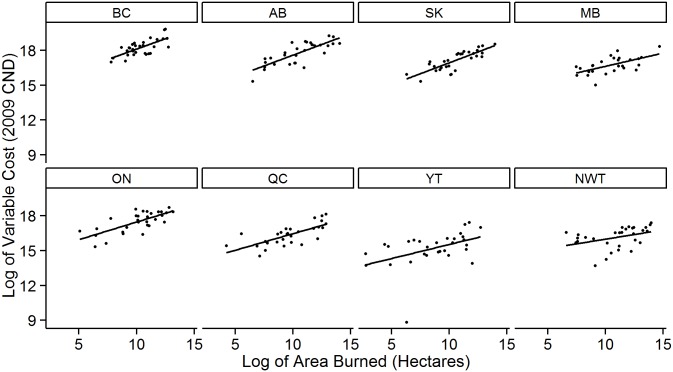
The relationship between the sum of area burned from May to August, and annual variable suppression costs, on a provincial basis.

The provincial area burned models show clear negative relationships between the natural logarithm of area burned and CMI (Fig 3). This relationship is strong in some provinces, and relatively weak in others, with an average provincial/territorial R^2^ value of approximately 30% (Table 1). All area burned models are statistically significant (*P* <0.05), with the strongest relationship found in British Columbia (R^2^ of 38%). The relationship between area burned and variable costs is consistently positive across provinces (Fig 4). All provincial models are statistically significant (*P* < 0.05), with an average R^2^ value of 42%; the model for Saskatchewan explains the most variation in variable costs with an R^2^ of 65% (Table 2).

**Table 1:** Provincial CMI and area burned linear regression model results.

Province	*β*_*0*_	*β*_*1*_	*F-Statistic*	*R*^*2*^	*p Value*
British Columbia	10.180	-0.171	19.030	0.383	0.000
Alberta	9.363	-0.165	5.437	0.133	0.027
Saskatchewan	9.461	-0.118	4.268	0.101	0.040
Manitoba	9.486	-0.240	15.850	0.373	0.001
Ontario	9.820	-0.250	17.020	0.356	0.000
Québec	11.347	-0.165	5.496	0.138	0.027
Yukon	4.614	-0.345	15.460	0.333	0.001
Northwest Territories	6.828	-0.373	15.560	0.334	0.000

**Table 2:** Provincial area burned and suppression cost linear regression model results.

Province	*β*_*0*_	*β*_*1*_	*F-Statistic*	*R*^*2*^	*p Value*
British Columbia	14.516	0.359	27.310	0.476	0.000
Alberta	13.880	0.370	47.200	0.614	0.000
Saskatchewan	13.169	0.376	53.920	0.646	0.000
Manitoba	14.342	0.228	13.540	0.334	0.001
Ontario	14.425	0.304	30.910	0.508	0.000
Québec	13.602	0.285	29.260	0.502	0.000
Yukon	13.103	0.243	6.810	0.167	0.014
Northwest Territories	14.384	0.160	4.750	0.115	0.038

### Projected area burned and costs

**Fig 5:**
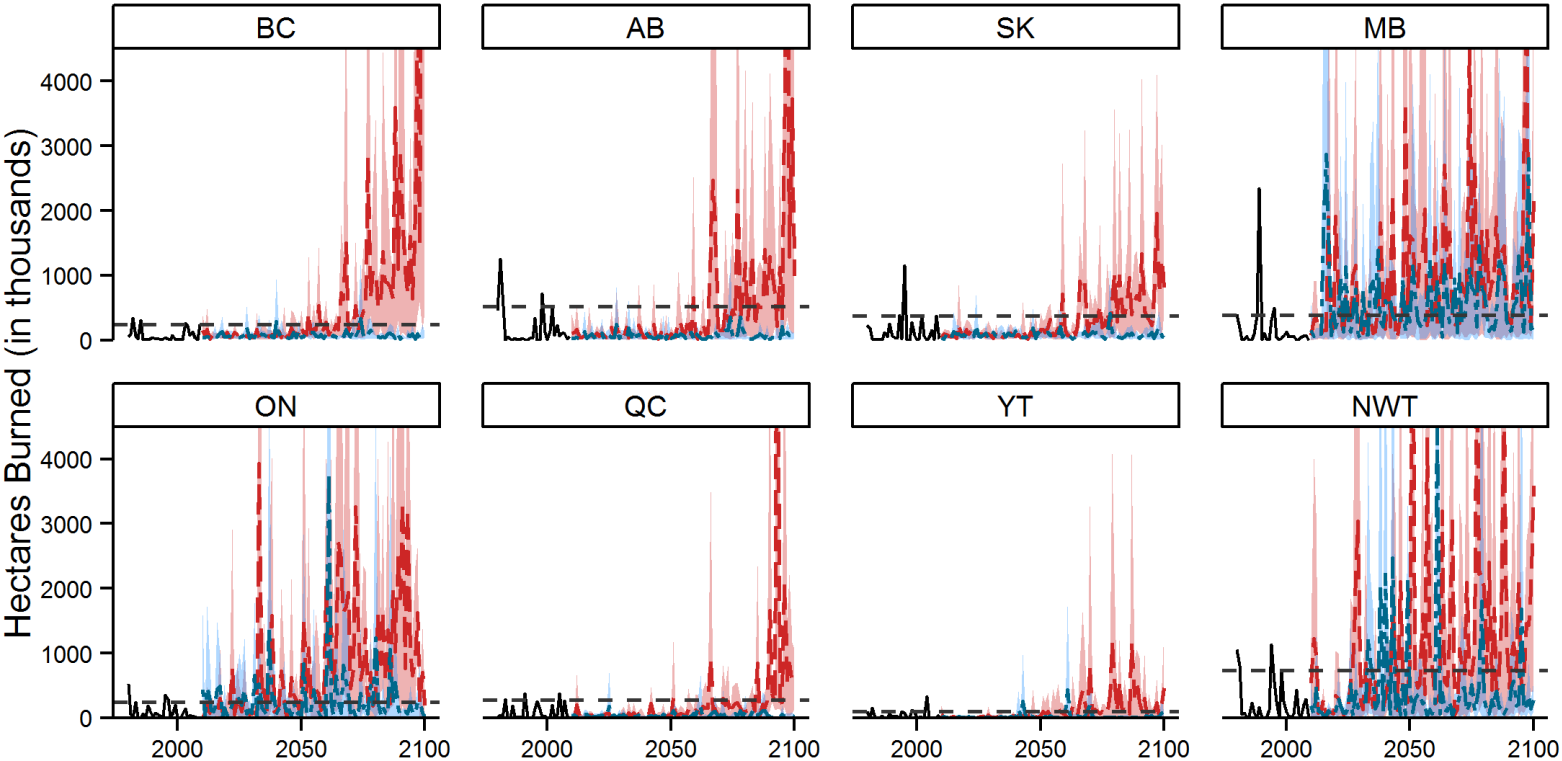
Area burned as forecast under RCP 2.6 and 8.5 for all GCMs. The blue and red shaded regions (RCP 2.6 and RCP 8.5 respectively) reflect the yearly maximum and minimum area burned estimates under the four GCMs; the model average is represented by the blue (RCP 2.6) or red (RCP 8.5) dashed line. The black dashed line represents the extreme fire year threshold, defined as the 90^th^ percentile value from the 1980–2009 period. The maximum area burned values for most provinces exceed the chosen scale and are not illustrated; British Columbia: 3.4x10^7^ ha in 2099; Alberta: 2.5x10^7^ ha in 2097; Manitoba: 1.4x10^7^ ha in 2048; Ontario: 1.5x10^7^ ha in 2033; Québec: 3.3x10^7^ ha in 2093; Northwest Territories: 2.9x10^7^ ha in 2051.

Under RCP 2.6, the provinces projected to experience frequent low CMI events (Manitoba, Ontario, and Northwest Territories) are also projected to experience frequent extreme fire events (Fig 5). These events are typically larger than historic fire events, and are projected to increase in size by the end of the century. Conversely, provinces and territories that are projected to experience less extreme fluctuations in CMI (Saskatchewan, Québec, and Yukon) are projected to experience little change in area burned. However, under RCP 8.5, all provinces and territories exhibit a distinct upward trend in area burned, with peaks regularly exceeding 10 million hectares in the second half of the century in most provinces. National summaries produce similar general patterns, with large increases in area burned through time and significantly larger areas burned under the RCP 8.5 scenario (Table 3).

**Table 3:** Area burned and suppression cost results for each GCM on a national scale.

GCM	Scenario		Average Annual Area Burned	Percentage Change	Average Proportion Burned^a^	Average Annual Total Suppression Cost^b^	Percentage Change
CanESM2		2011–2040	2.0 x10^6^	105%	0.61%	$ 7.7 x10^8^	25%
	**2.6**	2041–2070	2.1 x10^6^	113%	0.63%	$ 8.9 x10^8^	44%
		2071–2100	2.4 x10^6^	142%	0.72%	$ 1.1 x10^9^	72%
		2011–2040	1.9 x10^6^	96%	0.58%	$ 7.6 x10^8^	24%
	**8.5**	2041–2070	5.5 x10^6^	459%	1.66%	$ 1.0 x10^9^	69%
		2071–2100	1.1 x10^7^	1034%	3.37%	$ 1.5 x10^9^	141%
CESM		2011–2040	1.2 x10^6^	28%	0.38%	$ 7.0 x10^8^	13%
	**2.6**	2041–2070	1.9 x10^6^	99%	0.59%	$ 8.7 x10^8^	41%
		2071–2100	1.5 x10^6^	49%	0.44%	$ 1.0 x10^9^	62%
		2011–2040	2.0 x10^6^	105%	0.61%	$ 7.5 x10^8^	21%
	**8.5**	2041–2070	3.8 x10^6^	293%	1.17%	$ 9.8 x10^8^	59%
		2071–2100	7.5 x10^6^	665%	2.27%	$ 1.3 x10^9^	116%
HadGEM1		2011–2040	1.8 x10^6^	80%	0.54%	$ 7.4 x10^8^	20%
	**2.6**	2041–2070	1.6 x10^6^	66%	0.49%	$ 8.7 x10^8^	41%
		2071–2100	1.6 x10^6^	65%	0.49%	$ 9.9 x10^8^	60%
		2011–2040	2.0 x10^6^	109%	0.62%	$ 7.4 x10^8^	19%
	**8.5**	2041–2070	4.0 x10^6^	307%	1.21%	$ 9.5 x10^8^	55%
		2071–2100	1.1 x10^7^	977%	3.20%	$ 1.4 x10^9^	131%
MIROC		2011–2040	9.4 x10^5^	-4%	0.29%	$ 7.0 x10^8^	14%
	**2.6**	2041–2070	1.1 x10^6^	8%	0.32%	$ 8.6 x10^8^	40%
		2071–2100	9.5 x10^5^	-3%	0.29%	$ 9.9 x10^8^	61%
		2011–2040	6.1 x10^5^	-38%	0.18%	$ 6.9 x10^8^	13%
	**8.5**	2041–2070	2.9 x10^6^	195%	0.88%	$ 9.2 x10^8^	50%
		2071–2100	4.9 x10^6^	397%	1.48%	$ 1.2 x10^9^	89%
Average		2011–2040	1.5 x10^6^	52%	0.45%	$ 7.3 x10^8^	15%
	**2.6**	2041–2070	1.7 x10^6^	72%	0.51%	$ 8.7 x10^8^	39%
		2071–2100	1.6 x10^6^	63%	0.49%	$ 1.0 x10^9^	60%
		2011–2040	1.6 x10^6^	68%	0.50%	$ 7.4 x10^8^	19%
	**8.5**	2041–2070	4.0 x10^6^	313%	1.23%	$ 9.7 x10^8^	58%
		2071–2100	8.5 x10^6^	768%	2.58%	$ 1.4 x10^9^	119%

^a^Proportion of 328,488,862 hectares of the forested area of interest.

^b^Costs reported in 2009 Canadian dollars.

In an effort to present these temporal patterns in area burned in a more operationally meaningful way, we summarized the number of years in each future 30-year period (i.e., 2011–2040, 2041–2070, and 2071–2100) that fell into three fire activity categories. These categories were defined based on the area burned for each province from the recent historical period (1980–2009) and consisted of: 1) low fire years (area burned less than the 25^th^ percentile), 2) high fire years (area burned greater than the 75^th^ percentile), and 3) extreme fire years (area burned greater than the 90^th^ percentile). Full summaries are provided in Table 4; here we focus on extreme fire years—the cut-off for which is shown as a dashed line on each provincial plot in Fig 5. Note that, by definition, there were 3 extreme fire years for each province over the recent historical period. Clearly other definitions are possible and to some extent these thresholds are arbitrary but they support interpretation relative to today’s fire management context. Under RCP 2.6 there is little change in the frequency of extreme fire years, with only Manitoba (10 events) and Ontario (6 events) showing increases by the 2071–2100 period. Under RCP 8.5, all provinces are projected to experience an increase in the frequency of extreme fire years by the end of the century. At the low end of this increase, Québec is projected to experience an extreme fire year once every 3 years on average; at the high end, British Columbia and Ontario are projected to experience extreme years in two out of every three years. It is also noteworthy that low fire years, which currently occur 25 percent of the time, are projected to occur less than one percent of the time by the 2071–2100 period (Table 4).

**Table 4:** The number of years in which the estimated area burned achieves a specific threshold, reflecting low, high and extreme area burned years.

Area Burned			BC				AB				SK				MB				ON				QC				YT				NWT			
GCM	Scenario^a^		1	2	3	4	1	2	3	4	1	2	3	4	1	2	3	4	1	2	3	4	1	2	3	4	1	2	3	4	1	2	3	4
CanESM2		<25	8	1	4	2	8	6	7	7	8	1	1	3	7	1	1	0	8	5	2	1	8	6	7	4	8	1	1	2	8	3	4	4
	2.6	>75	8	14	10	14	8	7	4	8	8	7	4	6	7	22	22	22	8	15	12	18	7	1	5	7	8	0	5	1	8	10	10	14
		>90	3	4	4	4	3	1	0	1	3	0	1	1	3	9	11	11	3	10	11	9	3	1	3	3	3	0	3	1	3	5	3	1
		<25	8	2	2	0	8	4	5	1	8	2	2	0	7	1	0	0	8	2	0	0	8	5	2	0	8	1	0	0	8	6	1	0
	8.5	>75	8	16	22	28	8	8	12	23	8	4	14	22	7	21	18	22	8	11	26	28	7	3	12	23	8	0	11	17	8	9	21	22
		>90	3	4	12	25	3	3	6	17	3	0	5	18	3	9	12	15	3	6	20	22	3	1	9	18	3	0	8	14	3	5	18	15
HadGEM1		<25	8	2	0	3	8	4	2	9	8	0	1	1	7	2	3	3	8	4	4	8	8	7	6	12	8	1	0	0	8	3	0	0
	2.6	>75	8	10	10	11	8	4	3	3	8	4	3	4	7	13	19	18	8	13	9	6	7	0	1	0	8	0	0	1	8	10	15	15
		>90	3	1	0	1	3	1	0	1	3	2	0	1	3	6	9	13	3	12	4	2	3	0	1	0	3	0	0	0	3	2	5	5
		<25	8	1	0	0	8	5	4	0	8	2	2	0	7	2	0	0	8	8	3	0	8	7	3	0	8	0	0	0	8	1	0	0
	8.5	>75	8	13	20	30	8	4	6	21	8	4	7	23	7	19	20	22	8	9	14	21	7	1	0	14	8	0	2	20	8	13	16	20
		>90	3	0	5	28	3	0	2	8	3	0	4	15	3	9	9	12	3	7	9	18	3	0	0	8	3	0	2	18	3	4	11	17
CESM		<25	8	3	2	4	8	5	3	8	8	3	3	3	7	1	0	3	8	2	2	4	8	4	2	7	8	6	3	2	8	5	4	3
	2.6	>75	8	10	11	9	8	1	4	5	8	2	5	5	7	13	17	12	8	8	12	14	7	0	0	2	8	0	2	0	8	9	9	7
		>90	3	0	1	1	3	0	0	1	3	0	1	2	3	5	9	9	3	3	6	5	3	0	0	1	3	0	2	0	3	3	3	3
		<25	8	3	2	0	8	0	2	0	8	0	1	0	7	0	0	0	8	1	0	0	8	2	1	0	8	2	3	0	8	2	0	0
	8.5	>75	8	13	20	27	8	6	10	23	8	5	10	18	7	14	14	21	8	9	23	26	7	2	9	18	8	1	5	12	8	8	10	16
		>90	3	0	7	20	3	0	4	15	3	1	5	15	3	8	6	15	3	4	17	19	3	0	2	14	3	1	3	11	3	3	3	11
MIROC		<25	8	2	0	2	8	3	2	0	8	3	2	1	7	3	1	2	8	2	5	5	8	3	5	3	8	2	3	3	8	3	6	3
	2.6	>75	8	6	10	7	8	2	3	3	8	3	2	4	7	13	17	15	8	9	8	12	7	0	0	1	8	0	4	2	8	4	5	4
		>90	3	0	1	0	3	0	0	0	3	0	0	0	3	9	12	6	3	4	5	6	3	0	0	0	3	0	4	0	3	0	2	1
		<25	8	1	0	0	8	4	0	0	8	2	0	1	7	1	4	1	8	1	2	0	8	4	3	0	8	2	0	0	8	2	1	0
	8.5	>75	8	2	12	21	8	1	6	12	8	0	6	13	7	17	17	24	8	14	17	27	7	0	1	9	8	2	8	12	8	2	11	18
		>90	3	0	3	9	3	0	0	4	3	0	4	4	3	2	14	17	3	8	15	23	3	0	0	3	3	1	5	12	3	1	5	11
Average		<25	8	0	0	0	8	0	0	0	8	0	0	0	7	0	0	0	8	0	0	0	8	1	0	0	8	0	0	0	8	0	0	0
	2.6	>75	8	12	14	15	8	3	1	6	8	1	2	2	7	22	29	30	8	20	17	16	7	1	1	0	8	0	5	1	8	12	14	14
		>90	3	1	0	1	3	0	0	0	3	0	0	1	3	14	14	17	3	13	8	6	3	0	0	0	3	0	5	0	3	4	4	2
		<25	8	0	0	0	8	0	0	0	8	0	0	0	7	0	0	0	8	0	0	0	8	0	0	0	8	0	0	0	8	1	0	0
	8.5	>75	8	12	27	30	8	7	10	30	8	1	10	30	7	29	28	30	8	17	29	30	7	1	6	23	8	0	15	23	8	13	25	30
		>90	3	0	6	28	3	0	4	18	3	0	4	19	3	12	18	27	3	9	25	25	3	0	2	10	3	0	11	18	3	6	13	19

Thresholds examined include the number of years in which area burned was equal to or less than the 25^th^ percentile as defined in the 1980–2009 period, the number of years in which area burned was greater than the 75^th^ percentile defined from the base period, and the number of years in which area burned was greater than the 90^th^ percentile. The row of scenarios 1 through 4 reflects the time period, where scenarios 1, 2, 3, and 4 represent the 1980–2009, 2011–2040, 2041–2070, and 2071–2100 periods respectively.

^a^The area burned threshold levels (the 25^th^, 75^th^ and 90^th^ percentiles respectively) within each province are as follows; British Columbia (BC): 1.3 x10^4^, 7.3 x10^4^, 2.5 x10^6^; Alberta (AB): 1.1 x10^4^ 1.4 x10^5^, 5.2 x10^5^; Saskatchewan (SK): 1.2 x10^4^, 1.7 x 10^5^, 3.7 x10^5^; Manitoba (MB): 7.2 x10^3^, 1.0 x10^5^, 3.9 x10^5^; Ontario (ON): 7.4 x10^3^, 1.0 x10^5^, 2.5 x10^5^; Québec (QC): 5.5 x10^3^, 1.4 x10^5^, 2.7 x10^5^; Yukon(YT): 8.5 x10^2^, 6.8 x10^4^, 1.0 x10^5^; Northwest Territories (NWT): 1.3 x10^4^, 2.5 x10^5^, 7.4 x10^5^

Future variable costs under RCP 2.6 for all GCMs remain within the cost range established over the 1980–2009 period, although the year to year variation in costs increases in some provinces. In contrast, variable costs under RCP 8.5 rise significantly for all provinces other than the Yukon and the Northwest Territories. Based on the average of the four GCMs, annual variable costs in British Columbia, Alberta, and Ontario are projected to increase 79 to 145%, by the 2071–2100 period. The remaining provinces are projected to experience an average variable cost increase of 5–76% over the same period. Note that the nature of the composite model (i.e. average) smooths CMI values, thus reducing the variation exhibited by individual GCMs.

**Fig 6:**
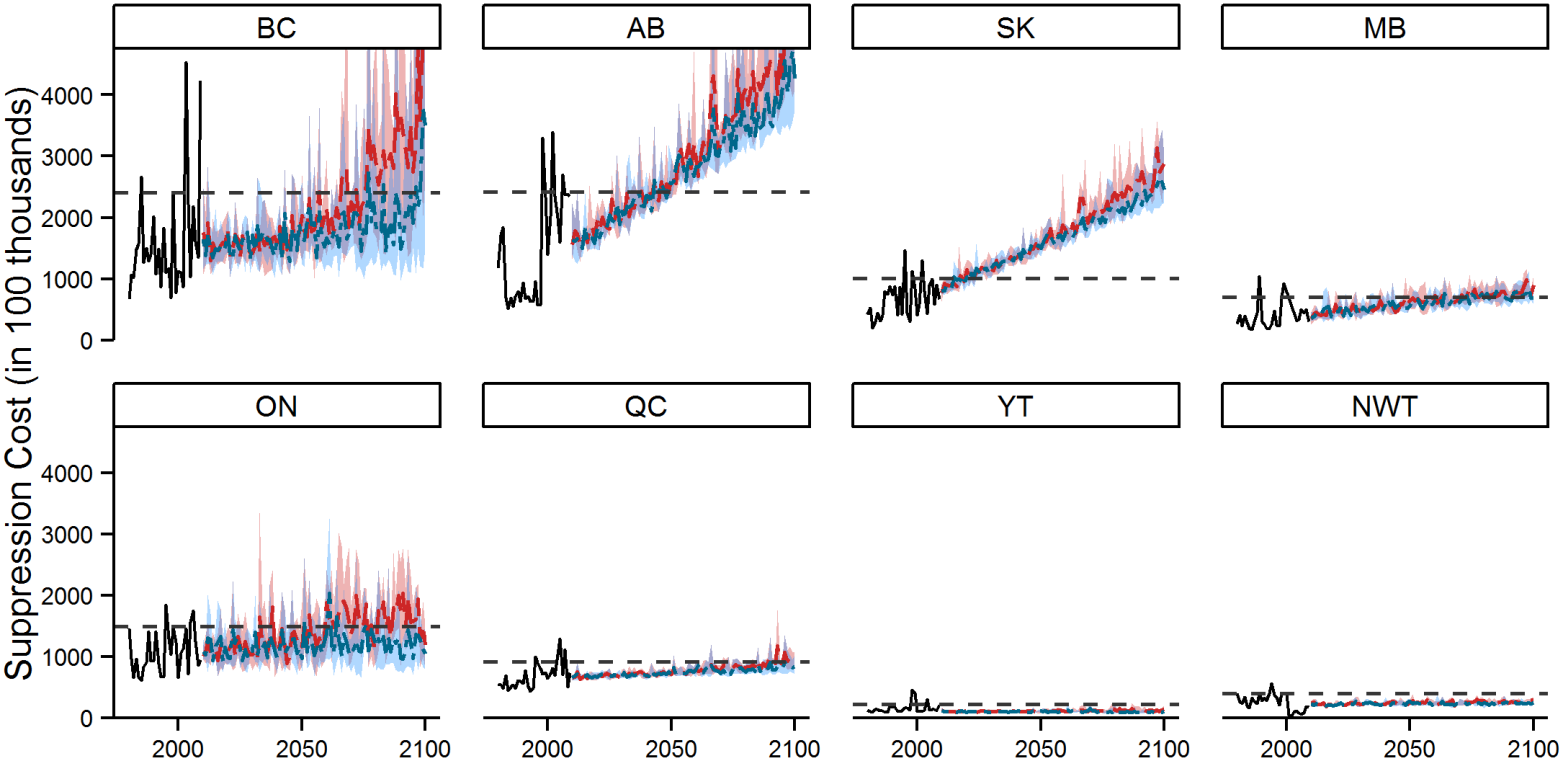
Total suppression costs as forecast under RCP 2.6 and 8.5 for all GCMs. The blue and red shaded regions (RCP 2.6 and RCP 8.5 respectively) reflect the yearly maximum and minimum total fire cost estimates under the four GCMs; the model average is represented by the blue (RCP 2.6) or red (RCP 8.5) dashed line. The black dashed line represents the extreme fire year threshold, defined as the 90^th^ percentile value from the 1980–2009 period. The maximum total cost values for Alberta and British Columbia are not illustrated within the figure; Alberta: $9.06x10^8^ in 2097; British Columbia: $1.07x10^9^ in 2099.

We also projected total costs by incorporating the fixed cost projections from the various autoregressive models (Table 5, Fig 6). Under RCP 2.6, annual average provincial total costs rise by 60–72% by the 2071–2100 period. This increase is greatest in Alberta and Saskatchewan, with costs rising by 141–218% by the end of the century. Alternatively, there are relatively minor changes in total costs in the Yukon, Northwest Territories and Québec, with end of century costs ranging between -42 and 13% of the base period costs. Note that a decrease in total costs results from a downward trend in fixed costs over the 1980–2009 period, which is then projected forward by the random walk with drift model. Under RCP 8.5, average provincial total costs are projected to increase significantly by the end of the century. Again, Alberta and Saskatchewan are associated with the largest increases, with average total costs rising by 195–265%, while the Yukon, Northwest Territories and Québec are projected to experience relatively small changes, ranging from a 19% decline to a 32% increase. National summaries indicate an overall increase in fire suppression costs through time, with total average annual costs exceeding $1.4 billion (2009 Canadian dollars) by the end of century under RCP 8.5 (Table 3).

**Table 5:** Provincial fixed cost autoregressive model results.

Province	Model Type	ARIMA (*p*,*d*,*q*)	AR 1	AR 2	AR 3	Drift	Intercept
British Columbia	Autoregressive with a constant value	(3,0,0)	0.315	-0.086	-0.602	-	5.2 x10^7^
Alberta	Random Walk with Drift	(0,1,0)	-	-	-	2.5 x10^6^	-
Saskatchewan	Random Walk with Drift	(0,1,0)	-	-	-	1.6 x10^6^	-
Manitoba	Random Walk with Drift	(0,1,0)	-	-	-	3.4 x10^5^	-
Ontario	Autoregressive with a constant value	(2,0,0)	1.019	-0.386	-	-	5.4 x10^7^
Québec	Random Walk with Drift	(0,1,0)	-	-	-	1.1 x10^5^	-
Yukon	Random Walk with Drift	(0,1,0)	-	-	-	-2.5 x10^4^	-
Northwest Territories	Autoregressive with a constant value	(1,0,0)	0.730	-	-	-	1.1 x10^7^

The number of low, high and extreme suppression cost years was calculated as described above for area burned (Table 6; see dashed line on Fig 6 for extreme cost cut-off value for each province). Provinces that showed a strong positive trend in suppression costs (Alberta, Saskatchewan, Manitoba) are projected to experience extreme suppression costs nearly every year by the end of the current century under both RCPs. Conversely, provinces/territories that showed little or no trend in suppression costs (Yukon, Northwest Territories) are projected to experience no extreme cost years in the future. It is noteworthy that both the Yukon and Northwest Territories have large suppression zones with minimal intervention, and thus are able to experience significant increases in area burned with little change in cost. The remaining provinces (British Columbia, Ontario, Québec) are projected to experience extreme costs roughly one out of every 2 years under RCP 8.5, but show little change in extreme year frequency under RCP 2.6.

**Table 6:** The number of years in which the estimated total suppression costs achieves a specific threshold, reflecting low, bad and extreme cost years.

Total Cost			BC				AB				SK				MB				ON				QC				YT				NWT			
GCM	Scenario^a^		1	2	3	4	1	2	3	4	1	2	3	4	1	2	3	4	1	2	3	4	1	2	3	4	1	2	3	4	1	2	3	4
CanESM2		<25	8	1	3	1	8	0	0	0	8	0	0	0	7	0	0	0	8	5	3	2	8	0	0	0	8	19	23	28	8	0	0	0
	2.6	>75	8	11	9	12	8	10	27	30	8	26	30	30	7	21	28	30	8	10	11	9	7	5	15	23	8	0	2	0	8	0	0	0
		>90	3	4	2	3	3	7	22	30	3	21	30	30	3	6	14	27	3	8	10	5	3	1	1	2	3	0	0	0	3	0	0	0
		<25	8	1	1	0	8	0	0	0	8	0	0	0	7	0	0	0	8	2	0	0	8	0	0	0	8	21	9	9	8	0	0	0
	8.5	>75	8	10	20	28	8	10	28	30	8	28	30	30	7	22	29	30	8	6	18	20	7	7	23	29	8	0	3	2	8	0	0	0
		>90	3	2	11	23	3	6	25	30	3	23	30	30	3	5	15	30	3	4	16	13	3	0	3	16	3	0	1	0	3	0	0	0
HadGEM1		<25	8	0	0	0	8	0	0	0	8	0	0	0	7	0	0	0	8	4	4	9	8	0	0	0	8	23	22	25	8	0	0	0
	2.6	>75	8	7	8	8	8	5	28	30	8	27	30	30	7	16	30	30	8	12	3	2	7	1	10	11	8	0	0	0	8	0	0	0
		>90	3	1	0	1	3	1	23	30	3	23	30	30	3	5	14	24	3	8	1	1	3	0	0	0	3	0	0	0	3	0	0	0
		<25	8	0	0	0	8	0	0	0	8	0	0	0	7	0	0	0	8	8	5	4	8	0	0	0	8	24	16	5	8	0	0	0
	8.5	>75	8	8	17	30	8	6	29	30	8	28	30	30	7	19	28	30	8	7	8	18	7	3	6	27	8	0	2	5	8	0	0	0
		>90	3	0	4	27	3	2	18	30	3	22	30	30	3	6	14	28	3	6	5	12	3	0	0	6	3	0	0	1	3	0	0	0
CESM		<25	8	2	1	2	8	0	0	0	8	0	0	0	7	0	0	0	8	4	2	6	8	0	0	0	8	24	24	30	8	0	0	1
	2.6	>75	8	5	7	4	8	2	27	30	8	26	30	30	7	16	29	30	8	3	5	4	7	6	13	20	8	0	1	0	8	0	1	0
		>90	3	0	0	1	3	1	24	30	3	17	30	30	3	3	11	21	3	2	2	4	3	0	0	1	3	0	0	0	3	0	0	0
		<25	8	1	1	0	8	0	0	0	8	0	0	0	7	0	0	0	8	2	1	0	8	0	0	0	8	17	19	15	8	0	0	0
	8.5	>75	8	9	17	26	8	8	28	30	8	28	30	30	7	18	28	30	8	4	15	17	7	8	19	30	8	0	2	2	8	0	1	1
		>90	3	0	5	20	3	5	25	30	3	22	30	30	3	7	10	28	3	3	13	14	3	0	0	13	3	0	0	0	3	0	0	0
MIROC		<25	8	0	0	1	8	0	0	0	8	0	0	0	7	0	0	0	8	2	6	5	8	0	0	0	8	17	20	25	8	0	0	0
	2.6	>75	8	2	6	1	8	3	28	30	8	24	30	30	7	15	30	30	8	3	5	4	7	4	10	18	8	0	1	0	8	0	0	0
		>90	3	0	0	0	3	3	23	30	3	18	30	30	3	5	14	26	3	2	4	2	3	0	0	0	3	0	0	0	3	0	0	0
		<25	8	0	0	0	8	0	0	0	8	0	0	0	7	0	0	0	8	1	2	0	8	0	0	0	8	19	14	16	8	0	0	0
	8.5	>75	8	1	7	17	8	0	30	30	8	26	30	30	7	20	27	30	8	6	15	22	7	4	11	30	8	0	1	5	8	0	0	0
		>90	3	0	1	7	3	0	25	30	3	20	30	30	3	0	16	28	3	1	12	19	3	0	0	2	3	0	0	1	3	0	0	0
Average		<25	8	0	0	0	8	0	0	0	8	0	0	0	7	0	0	0	8	1	0	1	8	0	0	0	8	24	25	30	8	0	0	0
	2.6	>75	8	4	5	2	8	5	29	30	8	27	30	30	7	22	30	30	8	4	4	2	7	0	10	26	8	0	0	0	8	0	0	0
		>90	3	0	0	0	3	1	24	30	3	19	30	30	3	0	14	30	3	1	2	0	3	0	0	0	3	0	0	0	3	0	0	0
		<25	8	0	0	0	8	0	0	0	8	0	0	0	7	0	0	0	8	0	0	0	8	0	0	0	8	20	9	4	8	0	0	0
	8.5	>75	8	3	20	30	8	6	30	30	8	29	30	30	7	23	30	30	8	2	16	24	7	3	22	30	8	0	0	0	8	0	0	0
		>90	3	0	4	26	3	1	25	30	3	23	30	30	3	1	21	30	3	2	10	22	3	0	0	9	3	0	0	0	3	0	0	0

Thresholds examined include the number of years in which total suppression cost was equal to or less than the 25^th^ percentile as defined in the 1980–2009 period, the number of years in which suppression costs were greater than the 75^th^ percentile defined from the base period, and the number of years in which suppression costs were greater than the 90^th^ percentile. The row of scenarios 1 through 4 reflects the time period, where scenarios 1, 2, 3, and 4 represent the 1980–2009, 2011–2040, 2041–2070, and 2071–2100 periods respectively.

^a^The total cost threshold levels (the 25^th^, 75^th^ and 90^th^ percentiles respectively) within each province are as follows; British Columbia (BC): 1.1 x10^8^, 1.8 x10^8^, 2.4 x10^8^; Alberta (AB): 6.9 x10^7^, 2.3 x10^8^, 2.4 x10^8^; Saskatchewan (SK): 4.2 x10^7^, 8.8 x 10^7^, 1.0 x10^8^; Manitoba (MB): 2.4 x10^7^, 4.4 x10^7^, 6.0 x10^7^; Ontario (ON): 8.4 x10^7^, 1.4 x10^8^, 1.5 x10^8^; Québec (QC): 5.1 x10^7^, 7.4 x10^7^, 9.3 x10^7^; Yukon (YT): 1.1 x10^7^, 1.7 x10^7^, 2.2 x10^7^; Northwest Territories (NWT): 1.7 x10^7^, 3.7 x10^7^, 4.0 x10^7^.

As noted, we also generated results using minimum fire return intervals of 10 and 30 years. Area burned decreased by 3% under RCP 2.6 and 20% under RCP 8.5 by the 2071–2100 period when the fire return interval was lengthened from 20 to 30 years, and increased by 0.05% under RCP 2.6 and 15% under RCP 8.5 when the fire return interval was shortened from 20 to 10 years. These changes in area burned translated into minor changes in costs, with total costs changing by less than 2% under the various fire return interval and RCP combinations. Thus our cost findings appear relatively robust to reasonable changes in the fire return interval employed here.

## Discussion

Our findings suggest that most Canadian provinces will experience significant increases in both area burned and suppression costs (in 2009 dollars) by the second half of the current century—particularly under RCP 8.5. For the country as a whole, annual suppression costs are projected to increase under RCP 8.5 by over 100% by the 2071–2100 period. To put these findings in context, for many provinces, annual costs that are currently considered extreme are projected to become commonplace by century’s end. Projections under RCP 2.6 were considerably less dire, providing another rationale for greenhouse gas mitigation efforts, as emission rates are currently tracking close to RCP 8.5 levels [47].

Several studies have explored the relationship between area burned and broad-scale climate indices. Price and Rind [55] used effective precipitation (a metric similar to CMI) to forecast area burned by lightning-origin fires in the United States; their model explained approximately 20% of the variation in observed monthly area burned. Similarly, Xiao and Zhuang [49] used the Palmer Drought Severity Index in a regression model to explain 24% of the variation in annual area burned across the boreal region of North America. Girardin and Wotton [56] reported an R^2^ value of 0.63 using a July drought index to model annual area burned for Canada over the 1959–99 period. However, this relatively high level of explained variance may have been influenced by the approach used to spatially average the drought index, in which a weighting scheme emphasized grid cells that were predetermined to have a strong relationship between annual area burned and the drought index (see [56] for details). In the current study, the strength of CMI as a predictor of area burned was comparable to these literature results but does vary considerably between provinces.

A number of factors may contribute to the mixed strength of the CMI-annual area burned relationships. First, area burned is an inherently stochastic metric as appropriate fire conditions need to be accompanied by ignition events in order for fires to occur. Furthermore, our CMI estimates represent broad spatial and temporal averages over a 4-month period within each fire suppression zone; this may tend to obscure fire behaviour relationships that are operating over finer spatial and temporal resolutions. Changes through time in fire reporting methodology, fire suppression zone boundaries, and/or fire management paradigms may add further noise to the area burned data. Québec had the highest (most wet) CMI values of any of the provinces in the study and the weak regression results for this province may indicate that the CMI-area burned relationship breaks down in relatively moist regions. In the case of Saskatchewan, the low R^2^ value may reflect the very high annual area burned in this province [17], such that natural feedbacks in the form of larger areas of younger stands with a higher deciduous component may be reducing fire frequencies and somewhat uncoupling the relationship between fire and climate.

Flannigan et al. [18] estimated an increase in area burned from 1.8 million hectares to between 3 and 4 million hectares annually for Canada by the end of the century under a 3 x CO_2_ emissions scenario (comparable to RCP 8.5). Our findings point to the possibility of a significantly higher annual area burned of 5.6 to 10.9 million hectares under RCP 8.5. Note that this difference would be even greater without our use of a minimum fire return interval, which was employed to constrain future annual area burned estimates based on reasonable ecological assumptions. There are a number of possible explanations for this disparity. Although both studies employ log transformed regression models, Flannigan et al. [18] used a different set of explanatory climate variables than those used here. Furthermore, rather than simply back-transforming logged projections of area burned, Flannigan et al. [18] expressed log transformed 3 x CO_2_ estimates as a ratio of log transformed 1 x CO_2_ estimates and obtained future values by multiplying historical area burned values by these ratios. In this way, they avoided the extremely large values that can arise when back-transforming logged projections of area burned; however, we question the appropriateness of multiplying unlogged historical values by ratios on a log scale. Finally, the studies cover slightly different land bases and employ different GCM versions, which may further contribute to the differences observed.

Our use of a 20-year minimum fire return interval was based on studies that report reduced fire probability in young boreal stands [54, 55]. As noted, the use of alternative fire return intervals (i.e., 10 and 30 years) resulted in moderate changes to area burned but had little impact on overall suppression costs—suggesting our results are relatively insensitive within this range of parameter values. This constraint reflects the negative feedback on area burned as larger and more frequent fires shift forest demographics and composition, particularly in the boreal, towards younger stands with higher deciduous components [42, 57]. It is noteworthy that in the absence of this constraint, our models predicted frequent, massive burns by the end of the current century in some regions. If realized, such a fire regime could involve a conversion from forests to grasslands, as the resulting fire cycle would likely be too short for trees to reach sexual maturity before being burned. Clearly such a conversion would have significant ecological and social implications, including major changes to fire suppression strategies and costs.

de Groot *et al*. [26] estimated that annual suppression costs could rise to between $656 and $760 million (in 2009 dollars) per year by the 2080–2100 period under a 3 x CO_2_ scenario. Our model projected higher end-of-century costs of $983 million under RCP 2.6 and over $1.4 billion under RCP 8.5. There are several noteworthy differences between these studies. First, de Groot *et al*. [26] developed their model using cost and area burned data over the 1970–1995 period; the data employed here is for the 1980–2009 period and includes several updates and improvements over the earlier dataset [2]. Note that we did not consider the 1970–1979 period in our analysis due to concerns about the accuracy of the area burned data over that period. Second, de Groot *et al*. [26] employ a preliminary version of the area burned estimates of Flannigan et al. [18], which, as described above, are considerably lower than those presented here. Finally, in order to simplify their approach, de Groot *et al*. [26] assumed a constant percentage increase in area burned each year, which ignores the influence of extreme fire years on estimated suppression costs.

A number of factors, not explicitly considered in our analysis, may add further pressure to wildfire response budgets. For instance, the length of the fire season is projected to increase over this century [11, 16], which may lead to larger area burned values (and attendant costs) than those estimated here. Similarly, increased fire intensities [58] may produce damage levels that are outside the scope of models calibrated using historical area burned-cost relationships. Population growth in the wildland-urban boundary [5], heightened public demand for protection of private property [59], and real increases in fossil fuel prices may also further increase fire suppression costs.

The projected increases in suppression costs presented here, particularly under RCP 8.5, may not be realized given competing demands for provincial budgets and the fact that recent studies show diminishing incremental returns on increasing expenditures [59]. If area burned does increase significantly, government agencies could be challenged to adopt fire management policies and practices that allow for more fiscally manageable responses [23, 26]. This could involve reducing the size of fire exclusion zones, responding to fewer fires, monitoring rather than aggressively attacking more fires, or re-evaluating suppression options after attacked fires have escaped initial attack; furthermore, the introduction of new technologies, such as an appropriate use of drones [60], could significantly alter the fire management landscape. While it is not possible to predict exactly how fire management will evolve over this century, our cost projections do provide a sense of the pressures likely to be placed on suppression budgets if alternative technology and policy approaches are not considered.

## Supporting Information

S1 Table Wildfire suppression costs for select provinces from 1980 to 2009. (CSV)
